# Prevalence and patterns of victimization and polyvictimization among female sex workers in Soweto, a South African township: a cross-sectional, respondent-driven sampling study

**DOI:** 10.1080/16549716.2017.1403815

**Published:** 2017-12-06

**Authors:** J. Coetzee, G. E. Gray, R. Jewkes

**Affiliations:** ^a^ Perinatal HIV Research Unit, University of the Witwatersrand, Chris Hani Baragwanath Hospital, Johannesburg, South Africa; ^b^ Department of Public Health, University of the Witwatersrand, Johannesburg, South Africa; ^c^ Office of the President, South African Medical Research Council, Cape Town, South Africa

**Keywords:** Sex work, South Africa, violence, client violence, intimate partner violence, hate crime

## Abstract

**Background:** Female sex workers (FSWs) are disproportionately affected by violence from multiple partner categories. This increases their vulnerability to HIV.

**Objectives:** To describe patterns of violence and polyvictimization among female SWs in Soweto.

**Methods:** A respondent-driven sampling (RDS) recruitment methodology was used to enrol 508 Soweto-based FSWs using a survey instrument. Raw and RDS adjusted data were descriptively analysed, Spearman’s correlation and chi^2^ test of association were used to show associations. Polyvictimization patterns are shown within a modified Venn diagram.

**Results:** The median age of FSWs in Soweto was 31 years, and most had an incomplete education (74.2%). The prevalence of exposure to physical/sexual intimate partner violence (IPV) in the past year was 53.8%, 46.8% by clients, and 18.5% by police. Past year prevalence of sexual/physical violence by any perpetrator category was 70.8% and lifetime exposure was 76.0%. Childhood sexual violence was reported by 44.3%. Lifetime non-partner rape was 55.5% and all rape exposure was 62.4%. As a result of engaging in sex work in the past year, 65.2% women had been discriminated against. Client, police, IPV, and childhood trauma were all significantly associated with one another, with IPV being the most common co-occurrence. Polyvictimization was seen in almost two-thirds of FSWs, and increased with exposure to discrimination.

**Conclusion:** In Soweto, FSWs are exposed to high rates of violence in multiple forms across their lifetime. Our findings show that violence continues unabated into adulthood at levels far higher than in the general population and overall at higher levels than previously recorded among SWs in South Africa. We argue that violence against FSWs is rooted in discrimination. The disparate burden of violence on FSWs requires urgent interventions to proactively address and reframe the normalisation of violence against all women.

## Background

Sex workers (SWs) epitomise South Africa’s dual human immunodeficiency virus (HIV) and violence epidemics [1–3]. Despite a constitution lauded internationally for being grounded in human-rights and equal rights for women [4], the country is highly patriarchal with pervasive violence against women [5]. Evidence confirms the high exposure female sex workers (FSWs) have to violence. A study of FSWs in Soweto, South Africa, showed that 53.8 and 55.6% had experienced intimate partner and non-intimate partner sexual violence in their lifetime (respectively). In Johannesburg, 20% of FSWs reported experiencing sexual violence, while 54% reported any physical violence in the past year [1,6]. Globally, violence is associated with inconsistent condom usage, sexually transmitted infection (STI) and HIV acquisition, undermining economic capital, and it has a widespread social impact [7–11]. The resulting mental health complications, including post-traumatic stress disorder (PTSD) and depression, often undermine individuals’ effective functioning [11,12]. This may result in substance abuse or a lack of adherence to chronic medications such as antiretroviral therapy (ART) [13]. For these reasons, it is important to understand exposure to violence and the multiple forms of violence FSWs may be exposed to across their lifetime to ensure that programmes can be effectively equipped. Little is known about the patterns and prevalence of victimization and polyvictimization among FSWs in South Africa . Given the launch of the *South African National Sex Worker HIV Plan 2016–2020*[14] and the scale up of sex work programs across the country, understanding the trauma experienced by this neglected and marginalised population is important to developing effective interventions.

South Africa’s socio-political history has fostered and normalised a culture of violence and inequality. The apartheid government sponsored violence, while state policies exposed millions of men and boys to humiliating, brutal, and violent police and prison practices. Alongside this there were limited and discriminatory resource allocations, poor education systems, and fragmented family structures, with institutionalised racism leaving a legacy of low self-worth. This violence was underpinned by notions of male superiority through intrinsically patriarchal values. One response among those most on the receiving end of apartheid violence, was the creation of more violent masculinities predicated on toughness, superiority, and sexual entitlement [15]. In contradistinction are models of respectable femininity that encourage subservience [5,16,17]. These masculinities persist to the present day, and male violence is still used to enforce male dominance and control, while punishing female transgressions [17–19]. Such relationship inequities result in greater vulnerability to HIV through increased and prolonged exposure to men who engage in violent and sexually risky behaviours. In a context where social norms discriminate against SWs and position them as a devalued population, this is compounded, while sex purchasers are not as socially condemned.

Evidence has shown the association between men who purchase sex and engage in IPV, rape, multiple concurrent partners, binge drinking, and refusal to use condoms [20–22]. They are also physically and sexually more violent towards women than are men who do not purchase sex [23], and men who engage in intimate partner violence (IPV) are more likely to be HIV positive [24]. This highlights the vulnerability of women selling sex to violent and often HIV positive men. Perpetration of violence by sex buyers is supported through a lack of emotional connection, stigma, and patriarchal entitlement [22,25]. Men desire relationships with women they respect [26]; however, SWs are not considered to be respectable women [27,28]. Furthermore, the stigma associated with sex work functions to increase the risk of IPV through the objectifying these women [29]. In South Africa, SWs describe being treated as if they were not human beings [30,31]. Together with patriarchal norms and aggressive masculinities, these factors function in important ways within a sex work–HIV epidemic. First, violence is used to punish women who transgress traditional gender norms [17,22], or who attempt to dictate the terms of a relationship and thus threaten male authority [19]. Second, SWs are not respected as women because they are seen to be ‘paid for’ [27], thus reducing male pride centring on sexual conquests, and positioning the FSW as being ‘owned’ by the paying partner. Finally, unlike a situation that can be found in intimate relationships, a sex exchange relationship offers no emotional protection from violence. These factors then result in perceived transgressions being punished through violence. SWs are not considered to have the right to give consent or to choose who they sell sex to, by clients, managers, police, and healthcare officials alike [6].

Data from a very deprived township north of Johannesburg CBD, South Africa, with similar conditions to those experienced by FSWs in Soweto, found that 38% of men reported perpetrating sexual assault, and 54% engaged in both physical and sexual violence in the past year [32]. These findings highlight the normalization of violence and that perpetrators feel impunity for their actions. For example, in Gauteng, more than half the women (51.3%) from the general population report experiencing some form of violence in their lifetime: 43.7% report emotional abuse, 33.1% physical violence and 23.5% sexual violence [33]. There is limited information available on exposure to violence for FSWs. The 2013 South African Health Monitoring Survey (SAHMS) asked two condensed questions about experiences of physical or sexual violence in the past year, finding physical and sexual violence to be 50.9% and 21.9%, respectively, among Johannesburg-based FSWs [1].

Experiences of violence in childhood are a known risk factor for later revictimization, including IPV [34,35]. The risk of IPV increases through witnessing violence against a mother [36], and experiencing sexual violence in childhood [11]. A 2013 study found a three-fold increase in gender-based violence among women who had experienced childhood violence [35]. Globally, evidence has also shown levels of childhood sexual abuse among FSWs to range from 32.4%–42.0%, and that sexual abuse in childhood is higher among SWs than other women [37,38]. Experiences of violence in childhood, normalize dysfunctional behaviors, and distort expectations of power. The trauma also affects the physiology of the brain, impacting upon emotional stability and relationship formation in adulthood, and promoting vulnerability to violence [11].

Evidence suggests that across South Africa, as many as one in eight women (13.8%) has experienced IPV in the past year [39]. In Soweto, 50.4% of women from the general population reported lifetime exposure to IPV and one-third reported IPV in the past year. Many women experience multiple violence types by an intimate partner [11]. Evidence suggests IPV is more common among women who report transactional sex [40]. Little is known about SW exposure to IPV in South Africa. One study found that 19% of SWs interviewed had been raped and 63% had been beaten by their intimate partner [41]. A Kenyan-based study found that 79% of FSWs had experienced some form of IPV in the preceding 30 days, which was associated with inconsistent condom use [42].

Some research exists on violence perpetration by police and clients of SWs in South Africa. In Cape Town 12% of FSWs reported ever having been raped and 47% ever threatened with violence by the police. Evidence suggests that SWs who attempt to report sexual violence to policing officials are frequently re-victimized by the police [6]. A 2011 qualitative study found that sexual violence perpetrated by police and clients was common [31]. Research has also found that 37% of street-based SWs and 20% of brothel-based SWs had experienced some form of client violence in their lifetime [6]. The vulnerability of SWs to violence is complex and requires more knowledge in developing an understanding of the patterns and prevalence of violence.

Modeling estimates have suggested that through addressing violence among SWs, 25% of HIV infections among this population could be averted [43]. Despite this, there is limited evidence on the (re-)victimization and polyvictimization of FSWs in South Africa. Available literature suggests that SWs are particularly vulnerable to violence perpetrated by clients and police. Yet, there is inadequate information on re-victimization by these perpetrator groups, nor the overlap with other forms of violence.

Our study aims to describe the prevalence of violence by perpetrator category and violence type, as well as patterns of victimization and polyvictimization, among FSWs in Soweto, South Africa. Although not directly comparable, we hypothesized that FSWs in Soweto experience higher levels of intimate partner violence and sexual abuse by any perpetrator than had previously been shown among women from the general population, and previously reported for FSWs in South Africa. Finally, we hypothesized that FSWs would experience substantially more childhood trauma than recorded for the general population.

## Method

Our cross-sectional study was conducted in Soweto. The locale is predominantly urban and peri-urban, low income with limited educational and employment opportunities. It has the highest population density in South Africa: an estimated two million inhabitants within >40 suburbs across 61km^2^. Eleven apartheid-era former single-sex and ethnically segregated hostels house an estimated 40,000 residents. A sex work program was launched by the Perinatal HIV Research Unit (PHRU) within the township in October 2013. Its fixed clinic was used as a base for the research [44]. Formative work included cognitive interviews (n = 12) and a pilot study (n = 40) [44,45]. The full methodology has been extensively described in previous research [45].

Inclusion criteria for the main study were: biologically female, over 18 years, currently sold sex in Soweto, knew their recruiter, and gave voluntary informed consent to participate. In determining sample size, a two-sided calculation was used to detect a difference between HIV prevalence among FSWs presumed to be exposed to violence versus those unexposed to violence. A sample size of 500 was estimated to ensure sufficient power of analysis.

A respondent-driven sampling (RDS) recruitment strategy was used [46]. The method is a popular way to recruit marginalized populations for population-level estimates [47,48]. A total of 11 seeds (initial participants) were used during the study to recruit 508 FSWs between February and September 2016. Similar to a chain referral method [47], all participants (including seeds) were given three coupons with which to recruit potential participants. Seeds and subsequently enrolled participants were asked to give the coupons to randomly selected women they knew and who knew them, who, like themselves, sold sex in Soweto, and who were older than 18 years. Recruitment chains were mapped between each seed and all subsequent recruits [48,49].

After screening, participants gave their consent to participate in the study, and then completed a 45-minute, interviewer-administered questionnaire in English, isiZulu, or SeSotho. Participants were then given a maximum of three uniquely coded coupons and reimbursed R100.00 ($7.69). A further R20.00 ($1.56) secondary incentive for successful recruitment was paid 7–10 days later. Primary reimbursement and secondary incentives were increased 5 months into the study to cover additional costs to participate [45].

Data were captured directly onto Lenovo tablets using the REDCap electronic data management system [50], hosted by the University of the Witwatersrand, South Africa. The database housed built-in skip patterns and algorithms. Duplicate data were collected on a convenience sample of 12% of the final sample size with an error rate of 0.6%. Ethical approval was provided by the Human Research Ethics Committee (Medical) of the University of the Witwatersrand, South Africa.

This study draws from an ecological model of understanding violence among SWs [51]. Various levels of influence exist within the environment, from the macrosystem which encompasses legal frameworks, and patriarchal and masculine identities, to the exosystem including the hostels, taverns, and brothels wherein sex is sold, and the health and policing systems. Finally, there is the individual level (micro system) experienced of violence perpetrated by clients, police, intimate partners, or experienced during childhood. These spheres of influence function to drive and maintain vulnerability to violence.

### Measures

Violence was assessed using the World Health Organization’s violence against women questionnaire [52] adapted to ask about violence specific to various perpetrator types. This included IPV, police violence, and client violence. Emotional violence was only asked in relation to intimate partners and included questions such as: ‘Within the past year did any partner insult you or make you feel bad about yourself?’, and ‘Within the past year did any partner threaten to hurt you?’ In assessing physical violence, questions included: ‘Within the past year did any [partner/client/police] hit you with a fist or with something else (such as a beer bottle, stick, or belt) which could hurt you?’ and ‘Within the past year did any [partner/client/police] kick, drag, beat, choke or burn you?’ For sexual violence, questions included: ‘Within the past year did any [partner/client/police] physically force you to have sex when you did not want to?’ and ‘Within the past year did you have sex with any [partner/client/police] when you did not want to because you were afraid of what he might do?’

All forms of violence were assessed by perpetrator category before moving on to the next perpetrator category. Questions were scored on a 4-point scale ranging from 1 ‘none’ to 4 ‘many’. Items were dichotomized as some versus no, to create seven new variables showing past year intimate partner emotional abuse, or physical/sexual violence by either an intimate partner, client, or the police. In addition, three variables indicating some or no physical/sexual violence were generated for intimate partners, clients, and police. The number of sexual assaults reported for each perpetrator category in the past year was used continuously. Any past year intimate partner emotional, physical, or sexual violence was generated and dichotomized. In addition, items were summed for each perpetrator category with scores >8 indicated some abuse and higher scores indicated increasing levels of abuse. Finally, all items were used to show past year and lifetime exposure to different types of violence (physical, sexual, and physical/sexual) irrespective of who the perpetrator was.

Other measures of violence included a shortened version of the childhood trauma questionnaire (CTQ) which measured five dimensions: neglect (physical and emotional) and abuse (emotional, physical, and sexual) [53]. Twelve questions were asked, including: ‘I saw or heard by mother beaten by her husband or boyfriend’, ‘I was insulted or humiliated by someone in my family in front of other people’, and ‘I had sex with someone who was not my boyfriend because I was threatened or frightened or forced.’ Due to a lack of variability, items were scored on a 4-point scale ranging from 1 ‘never’, 2 ‘rarely’, 3 ’many’ and 4 ‘very often’. Items were dichotomized to create a ‘none/rarely’ and ‘many/very often’. Items were also summed, with scores >12 indicating some abuse and higher scores indicating increasing levels of abuse. In our study the CTQ yielded a Cronbach alpha of 0.77, suggesting good reliability. Data were also dichotomized to show high levels of emotional and physical abuse and neglect. An additional question on first sex was added to show some/none sexual abuse: ‘Which of the following statements most closely described your experiences the first time you had sexual intercourse? I was willing, persuaded, tricked, forced, or raped.’ For this item, non-consensual sex was defined as being ‘tricked, forced, or raped’.

Overall, internal and external stigma were measured using an adapted version of the Stigma Index. Questions to measure external stigma included: ‘Within the past year have you been beaten or threatened because you are a sex worker?’ and ‘Within the past year have you been denied health services because you are a sex worker?’, with responses being on a 4-point scale of 1 never to 4 often. Questions to measure internalization of stigma included: ‘Within the past year I have felt ashamed because I am a sex worker?’, and ‘Within the past year I have felt guilty because I am a sex worker.’ Items were scored as ‘strongly agree’ to ‘strongly disagree’. A variable was generated which indicated that stigma has been experienced and whether it was only internal, external, or a combination of both. A second stigma variable was developed which indicated some or no stigma, but excluded stigma related rape to avoid a duplication of item use between this and polyvictimization.

In assessing other forms of sexual assault, stigma-related rape was measured using one question: ‘Within the past year have you experienced sexual abuse because you are a sex worker?’ and gang rape: ‘Have you ever experienced a gang rape?’ (rape by multiple perpetrators). Both were scored on a 4-point scale of 1 ‘never’ to 4 ‘very often’, and dichotomized as none versus some. We also asked: ‘How often has this [gang rape] happened?’ (continuous). Two additional variables were then generated: any non-intimate partner rape ever reported across the study, which included client, police, stigma, childhood, and gang-related rapes. The second was all rapes ever reported and included intimate partner rape.

To show the overlap between different forms of violence, and using the afore mentioned variables generated to show some or no violence by a perpetrator category or during childhood, new dummy variables were created which showed whether or not two kinds of violence had been experienced (e.g. childhood and IPV, or childhood and client violence). All possible combinations of violence experienced by perpetrator categories or during childhood were then generated. These were used to create a Venn diagram of polyvictimization. In addition, and to show how many types of violence an individual had experienced across their lifetime, a variable was generated to showing incremental increases in exposure to the dichotomized variables showing childhood trauma, IPV, police, or client violence.

Coupon numbers were recorded and linked between seeds, recruiters, and subsequent recruits. Participants were asked a three-part question to obtain their network size [45]. RDS weighting used for the adjustment was based upon each participant’s relative network size and recruitment chains. Population-level estimates were developed using crude sample data adjusted to reflect the target population based upon RDS weights. Socio-demographic characteristic items were used as single items. Questions including: date of birth ‘What date were you born?’, home language ‘What language do you speak?’ (all 11 official languages were listed as well as ‘other’), and place of birth (all South African provinces were listed, as were countries with whom South Africa shares a border), and highest level of education achieved. Participants were asked about whether or not they were currently dating a male or female partner, the age of their current partner – ‘How old is your current intimate partner?’, the number of intimate partners (main partners) they had had in the past year ‘How many intimate partners [non paying] have you had in the past year?’, and whether or not their partner was employed.

### Statistical analysis

**Figure 1.**
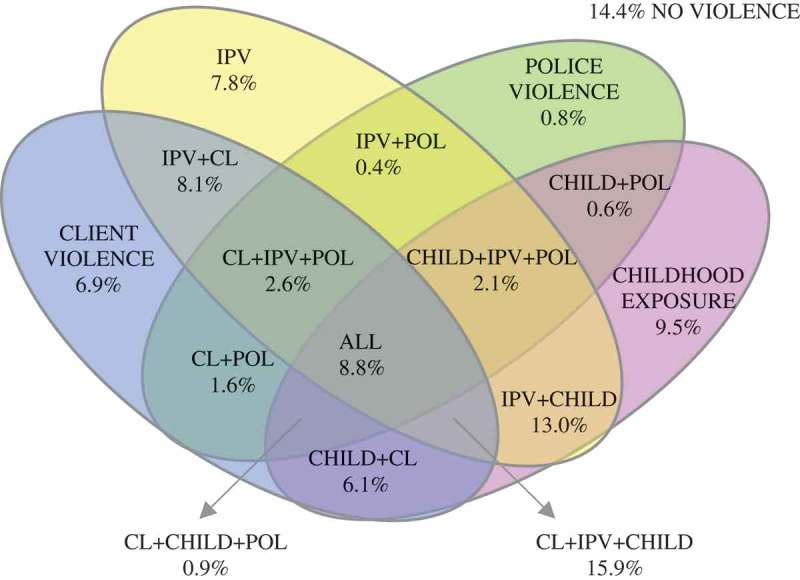
Victimization and polyvictimisation between IPV, client and police violence, and childhood exposure to violence (RDS adjusted %).

In analysing RDS data, specialized statistical software (Respondent-Driven Sampling Analysis Tool [RDSAT] [54]) was used to assess RDS assumptions including homophily and convergence, and to produce RDS-II population-level estimates [44]. The program is freely available for download along with an operational manual (http://www.respondentdrivensampling.org). Univariate descriptive statistics and violence prevalence were determined (Tables 1 and 2). Spearman’s correlation was used to develop a covariance matrix of continuous measures for IPV, client violence, police violence, and CTQ using only unadjusted data (Table 3). Victimization and re-victimization patterns are shown within a modified Venn diagram (Figure 1) and a chi^2^ test of association between the number of overall violence categories experienced and discrimination (Table 4). Analysis of data was done using RDSAT (adjusted) and STATA13 (unadjusted).

**Table 1.** Description of demographic characteristics of female sex workers in Soweto, including crude and RDS adjusted percentages with 95% CI.

	Overall	
Variable (n = 508)	n (%)	Adj % (95% CI)^a^
**Demographic characteristics**		
Age median (MED, IQR)^b^	30 (18–59)	31 (25–37)
Home language		
Zulu	274 (53.9)	52.3 (46.0–59.5)
Sotho	131 (25.8)	26.7 (20.5–32.5)
Other	103 (25.8)	21 (15.9–26.2)
Place of birth		
Gauteng	346 (68.1)	71.6 (64.6–77.2)
KwaZulu Natal (KZN)	86 (16.9)	13.1 (9.4–17.9)
Other	76 (15)	15.2 (10.5–20.3)
Education		
Incomplete schooling	384 (75.6)	74.2 (69.2–79.9)
Secondary complete/Some tertiary	124 (24.4)	25.8 (20.4–30.9)
Number of intimate partners (past year) (MED, range)	1 (0–20)	1.0 (1.0–1.0)
Number of FSW with current male partner	498 (98.0)	97.1 (94.2–99.1)
Age of intimate partner (MED, range)	34 (20–62)	34 (28–40)
Employment status of intimate partner		
Unemployed	142 (27.9)	30.7 (25.5–35.8)
Employed	366 (71.1)	69.3 (64.2–74.5)

^a^Adjusted % (95% confidence interval) (Adj % (95% CI)

^b^Median, interquartile range (MED, IQR)

**Table 2.** Prevalence and perpetrators of violence, including a summary of overall sexual assault reported, for FSWs in Soweto, including raw and RDS adjusted percentages and CI.

	n(%)	Adjusted % (95% CI)	n(%)	Adjusted % (95% CI)	n(%)	Adjusted % (95% CI)	n(%)	Adjusted % (95% CI)
**Violence by perpetrator & type**	**Intimate partner**		**Client**		**Police**		-	-
Emotional (12 months)	333(65.6)	63.3(57.2–68.2)	-	-	-	-	-	-
Physical (12 months)	268(52.8)	48.6(43.7–54.3)	247(48.6)	42.7(38.1–48.6)	38(7.5)	7.2(4.0–10.7)	-	-
Sexual (12 months)	105(20.7)	20.4(16.0–25.1)	74(15.6)	13.8(10.1–17.8)	20(3.9)	3.6(1.8–5.5)	-	-
Median number of sexual assaults MED(IQR)	3(1–30)	3.0(1.0–5.0)	1(1–7)	1.0(1.0–3.0)	1(1–6)	1.0(1.0–1.5)	-	-
Sexual ever	124(24.4)	24.4(19.6–29.3)	91(17.9)	16.9(13.1–21.0)	30(5.9)	5.1(2.9–7.4)	-	-
Physical/Sexual violence (12 months)	288 (56.7)	53.8(48.1–51.9)	265(52.2)	46.8(41.8–53.0)	53(10.4)	18.5(14.2–23.7)	-	-
Any intimate partner violence (12 months)	353(69.5)	67.4(61.3–72.7)	-	-	-	-	-	-
**Overall violence type by perpetrator: 12 months and ever reported**	**Physical**		**Sexual**		**Physical/Sexual**		**Overall**	
Any by perpetrators (12 months)	366(72.1)	67.6(62.8–73.2)	161(31.7)	30.4(25.5–35.7)	379 (74.6)	70.8(66.2–76.0)	416(81.9)	79.3(74.6–84.1)
Any by perpetrators (Ever)	386(76.0)	72.9(67.8–78.5)	199(39.2)	38.0(32.2–43.4)	399 (78.5)	76.0(71.2–81.3)	430(84.7)	82.3(78.2–86.9)
**Childhood abuse**	**Physical**		**Sexual**		**Neglect**		**Emotional**	
Neglect	102 (20.7)	20.8(16.4–25.5)	79(15.6)	14.8(11.1–18.2)	93(19.3)	17.2(13.2–22.0)	213 (41.9)	44.3(38.6–50.1)
Childhood abuse scale (MED, Range)								
**Exposure to stigma**	**NO STIGMA**		**Internal stigma**		**External stigma**		**Both forms**	
No stigma experienced	85(16.7)	17.7(13.5–22.5)	75(14.8)	17.1(12.4–21.7)	54(10.6)	10.0(7.3–13.7)	294(57.9)	55.2(49.0–60.5)
**All other sexual violence reported**	**Stigma-related rape**		**Gang raped ever**		**Non-intimate partner rape (ever)**			
Sexual violence	55(10.8)	8.4(5.7–11.3)	25(4.9)	4.5(2.5–7.1)	277 (54.5)	55.5(50.3–61.3)	314(61.8)	
Number of gang rapes MED(IQR)	1(1–6)	1.0(1.0–1.0)	-	-	-	-		-

**Table 3.** Covariance matrix of violence scores by perpetrator type and CTQ.

	MED^a^(Range)	IPV	Client violence	Police violence	CTQ
IPV^b^	7 (6–24)	1			
Client violence	7 (6–17)	r_s_ = 0.21, p = < 0.001	1		
Police violence	6 (6–10)	r_s_ = 0.16, p = 0.003	r_s_ = 0.24, p = < 0.001	1	
CTQ^c^	18 (13–31)	r_s_ = 0.28, p = < 0.001	r_s_ = 0.20, p = < 0.001	r_s_ = 0.17, p = < 0.001	1

^a^Median (MED)

^b^Intimate partner violence (IPV)

^c^Childhood trauma questionnaire (CTQ)

**Table 4.** Polyvictimization across the lifetime of FSWs in Soweto (IPV, client violence, police violence, and childhood exposure to violence) by exposure to discrimination (community, familial, police, or health official).

	Overall polyvictimization only		Some external discrimination by polyvictimization		No external discrimination by polyvictimization	
Polyvictimization	N(%)	Adj % (95% CI)	N(%)	Adj % (95% CI)	N(%)	Adj % (95% CI)
No violence	69 (13.6)	14.4 (10.3–18.6)	34 (9.9)	**11.3 (6.4–16.6)**	35 (21.5)	**20.5 (13.4–27.9)**
1 type	117 (23.0)	24.3 (19.3–29.5)	66 (19.1)	20.5 (15.2–26.1)	51 (31.2)	31.3 (21.9–41.8)
2 types	158 (31.1)	31.0 (25.4–35.7)	105 (30.4)	31.7 (24.7–37.5)	53 (32.5)	29.8 (20.3–39.2)
^3^3 types	164 (32.3)	30.2 (25.5–36.4)	140 (40.6)	**36.5 (30.4–44.1)**	24 (14.4)	**18.5 (10.8–28.2)**

## Results

Table 1 shows that the median age of FSWs in Soweto was 31 years, and 71.6% from the Gauteng province. Most had incomplete schooling (74.2%) and 97.1% had a current male intimate partner.

The most commonly reported form of violence was past year IPV, reported among 63.3% (emotional), 48.6% (physical), and 20.4% (sexual) of the FSWs. The median number of sexual assaults by an intimate partner was three (1–30), with 24.4% of FSWs reporting lifetime exposure to intimate partner rape. Client violence was the second most commonly reported form of violence, with physical and sexual violence in the past year being reported by 42.7% and 13.8%, of FSWs, respectively. Any physical or sexual violence by a client in the past year was reported by 46.8% (Table 2).

Analysis of violence types by all three perpetrator categories combined (Table 2) shows that any physical violence perpetrated by any of the three perpetrators in the past year was 67.6%, while any violence ever by a perpetrator was 72.9%. The prevalence of any sexual violence perpetrated by any of the three perpetrators, in the past year, was 30.4%, while 38.0% reported ever experiencing sexual violence by any of the three perpetrators. The prevalence of any violence reported for all three perpetrator categories was 79.3% in the past year and 82.3% ever. The childhood trauma questionnaire showed that sexual violence was experienced by 44.3% of FSWs. Both internal and external stigma were reported by 55.2% of FSWs. All non-intimate partner rape ever reported was 55.5%, and overall 62.4% ever reported a rape.

Figure 1 shows overlaps in victimization of FSWs in Soweto, with only 14.4% experiencing no childhood, police, client, or intimate partner violence across their lifetime. The greatest proportion of overlap is seen between IPV, and client violence and childhood trauma among 15.9%, or childhood trauma with 13.0% of FSWs.

Table 4 shows that 24.3% experienced only one form of violence. Two forms of violence were experienced by 31.0% of FSWs and three of more by 30.0% of FSWs in Soweto. The number of FSWs experiencing multiple forms of violence per category increased significantly for those who had experienced discrimination as compared with those who had not: 11.3% no violence, 20.5% one exposure, 31.7% two exposures and 36.5% three or more exposures.

## Discussion

Our findings show the disparate vulnerability of FSWs in Soweto to violence by multiple partner categories and across the lifetime of FSWs. We hypothesized that FSWs in Soweto, South Africa, experience higher levels of intimate partner violence and sexual abuse by any perpetrator than had previously been shown among women from the general population. We found that almost 90% of FSWs had experienced some form of violence across their lifetime, highlighting their immense vulnerability. Lifetime likelihood of experiencing sexual violence was almost two-thirds for FSWs, which is 2.6 times higher than the prevalence reported for women in the general population of Gauteng (62.4% versus 23.5%) [33], and suggests an overwhelming exposure to rape and other forms of sexual violence for FSWs in Soweto. We were correct in our hypothesis that childhood sexual violence exposure would be higher among FSWs as compared to the general population in South Africa (44.3% vs 35.4%) [55]. This is consistent with previous findings that childhood sexual assault is higher among SWs [38]. Together these findings suggest that engaging in sex work is a key aspect of vulnerability to the high levels of violence in adulthood and denotes the relationship inequalities between FSWs and their partners (both paying and non-paying).

As hypothesized, our findings were similar but with higher levels of reporting exposure to violence by type, as compared to other studies of violence exposure in FSWs. Sexual violence in the preceding year was almost 50% higher than that recorded in the SAHMS (30.4% vs 21.9%). However, this is to be expected given the depth of questioning within our study, which is not included in other studies on violence among FSWs. Exposure to IPV was similar to a 2015 study on violence against substance abusing SWs [41]. The unabated continuation of violence into adulthood resulted in two-thirds of FSW experiencing rape and a more than three-quarter lifetime exposure to other forms of violence. These findings show the importance of gaining a comprehensive understanding of violence in FSW populations where we have shown substantially higher prevalence overall than previously thought. Given the aggressive masculinities prevalent in South Africa, that FSWs transgress a multitude of social norms, and that sex is paid for, SWs are positioned as a commoditized and dehumanized population. Their relationships with intimate partners, clients, and the police are prone to being inequitable, with physical and sexual violence against SWs possibly being perceived by the perpetrators as a ‘justifiable violence.’ Thus, there are higher levels of violence among FSWs than perpetrated against women from the general population.

When we consider violence by perpetrator category, we found that prevalence varied between our and previous research. Exposure to intimate partner physical violence was lower in our study as compared with substance abusing SWs (48.6% vs 63.3%) [41]. When compared to Kenya [42], our findings were lower (53.8% vs 79%). However, the prevalence of intimate partner rape was consistent with previous research (20.4% vs 19%). Client physical and sexual violence (combined) in the past year was higher in our study than in other studies, with almost half of Sowetan FSWs having experienced physical or sexual violence (46.8%) compared with 37 and 20% of street- and brothel-based SWs in Cape Town. Furthermore, and in relation to all forms of violence, we show that there is little variance in exposure to violence by perpetrators for the past year as compared with ever exposed. This highlights the consistent exposure to violence which FSWs are exposed to. These findings, although abhorrent, are unsurprising given the aggressive masculinities that SWs can be exposed to. We are concerned that violence perpetration with impunity functions as a driving factor in this epidemic and is possibly magnified in township settings.

Female sex workers are more vulnerable to the co-occurrence of violence than women in the general population. More than 50% of polyvictimization involved IPV, which highlights the vulnerability to violence by both non-paying and paying partners. That discrimination was strongly associated with the co-occurrence of violence provides important information on the nature of violence against FSWs. This suggest that violence against FSWs is a systematic, work-related discrimination, making it a hate crime. The men who purchase sex are more likely to engage in other violent and criminal activities [25], resulting in an inherent vulnerability for FSWs to violent partners, both clients and intimate. Furthermore, hostilities toward SWs are often grounded in moral ideologies surrounding gender normative behaviors which SWs transgress. Perpetrators of violence against SWs are infrequently held accountable. This is underpinned by a legislative framework criminalizing SW in South Africa, which enables unlawful and criminal actions against SWs by policing officials. Violence by police against SWs in South Africa has been well documented [56–59], and while our study shows lower levels than previous recorded among SWs in South Africa, it nonetheless adds to a growing body of evidence highlighting police violence and brutal treatment of SWs.

South Africa is currently in the process of scaling up HIV prevention services for SWs, using the National Sex Worker HIV Plan 2016–2020 as a framework [14]. While these guidelines strongly encourage comprehensive services geared to addressing GBV, it is unclear what this means in terms of interventions in this context. The implementation of a strategy to guard SW rights, while a notable step in the right direction, does require structured programs to address the prevention of GBV, and address the gender norms underpinning violence against FSWs.

Our study had several limitations. Violence perpetrated by police and gang rape were possibly underreported and may be due to their order in the survey document (asked after client and intimate partner experiences) that could have resulted in participants feeling emotionally overwhelmed and unable to disclose further violence exposure. Other forms of violence were not asked about in this study, including police neglect, and violence by family members and strangers. As such we feel that our findings are an underreporting of violence prevalence. The study focused exclusively on FSWs, with no clients included. There was some variation between adjusted and unadjusted analysis. Weights were not included in the covariance matrix. Future research is required which aims to understand perpetrator specific patterns of violence and associated factors to ensure that SW program implementers can address areas of concern. Furthermore, research should understand the impact of violence on mental health and on adherence to HIV-related medications.

## Conclusion and policy recommendations

The intersection between gender inequality, poverty, violence, and HIV [60] creates a public health crisis centering on sexual and physical violence. Sex workers have a heightened vulnerability to violence with the prevalence of polyvictimization confirming that they are positioned on the apex of South Africa’s broader violence epidemic. The power differential between FSWs and the men with whom they interact intensifies their vulnerability. The current legislative framework functions to legitimize violence against SWs through endorsing discrimination and thereby encouraging ideas that there could be ‘justifiable’ or ‘deserved’ violence against SWs. The South African government needs to take heed of the growing body of knowledge highlighting the vulnerability of this population and begin safeguarding their human rights as part of addressing the overall violence epidemic in South Africa. It is imperative that legislation criminalizing SW be reviewed as a fundamental mechanism to addressing violence against SWs. Exposure to such high levels of violence are likely to hamper HIV prevention and treatment interventions targeting FSWs. Thus, there is an urgent need for a program of research to define what works to prevent violence against SWs from paying and non-paying partners, and clearly define and implement appropriate and inclusive sex work sector violence prevention interventions. Taken together, our findings suggest the normalization of violence against SWs, which requires collective attention by policy makers, program implementers, researchers, and community members.

## Figures and Tables

**Table:** 

Literature known	SW are exposed to high levels of violence by client and police in South Africa. Little is known about the co-occurrence of violence across their lifetime
Gaps filled	Exposure to violence is extremely high and co-occurring across their lifetime, with intimate partners being major perpetrators. Past year violence exposure is almost as high as lifetime exposure, suggesting the consistent reoccurrence of violence
Implications	Interventions targeting male sex partners and addressing gender inequality need to be urgently developed and implemented concurrently to sex work programs in South Africa.
